# The victims of unethical human experiments and coerced research under National Socialism

**DOI:** 10.1016/j.endeavour.2015.10.005

**Published:** 2016-03

**Authors:** Paul Weindling, Anna von Villiez, Aleksandra Loewenau, Nichola Farron

**Affiliations:** 1 Oxford Brookes University, History, Philosophy and Religion, Headington Campus, Oxford OX3 0BP, United Kingdom; 2 Independent Data Analyst; 3 Oxford Brookes University, Centre for Medical Humanities, Department of History, Philosophy and Religion, Gypsy Lane, Oxford OX3 0BP, United Kingdom; 4 University of Calgary, Cumming School of Medicine, 2500 University Drive NW, Calgary T2N 1N4, Canada; 5 Independent Holocaust Historian

**Keywords:** Unethical human experiments, German National Socialism, Holocaust, Jewish victims, Gypsy victims, Mengele, Auschwitz, Concentration camps, Nazi euthanasia

## Abstract

There has been no full evaluation of the numbers of victims of Nazi research, who the victims were, and of the frequency and types of experiments and research. This paper gives the first results of a comprehensive evidence-based evaluation of the different categories of victims. Human experiments were more extensive than often assumed with a minimum of 15,754 documented victims. Experiments rapidly increased from 1942, reaching a high point in 1943. The experiments remained at a high level of intensity despite imminent German defeat in 1945. There were more victims who survived than were killed as part of or as a result of the experiments, and the survivors often had severe injuries.

## Highlights

• Human experiments were more extensive than often assumed with a minimum of 15,750 documented victims.
• Experiments rapidly increased from 1942, reaching a high point in 1943 and sustained until the end of the war.
• There were more victims who survived than were killed as part of or as a result of the experiments. Many survived with severe injuries.
• Victims came from diverse nationalities with Poles (Jews and Roman Catholics) as the largest national cohort.
• Body parts, especially from euthanasia killings, were often retained for research and teaching after 1945.

## Background

The coerced human experiments and research under National Socialism constitute a reference point in modern bioethics.^7^ Yet discussions of consent and the need for safeguards for research subjects to date lack a firm basis in historical evidence. There has been no full evaluation of the numbers of victims of Nazi research, who the victims were, and of the frequency and types of experiments and research. The one partial estimate is restricted to experiments cited at the Nuremberg Medical Trial. This paper gives the first results of a comprehensive evidence-based evaluation of the different categories of victims. In 1945 liberated prisoners from German concentration camps began to collect evidence of the experiments.

The scientific intelligence officer John Thompson then pointed out not only that 90% of all medical research under National Socialism was criminal, but also the need to evaluate all criminal experiments under National Socialism, and not just those whose perpetrators were available for arrest and prosecution.^8^ The Nuremberg Medical Trial of 1946–47 was necessarily selective as to who was available for prosecution, and since then only clusters of victims have been identified.^9^ In the early 1980s Günther Schwarberg named a set of child victims: his reconstruction the life histories of the ‘twenty children’ killed after transport from Auschwitz for a tuberculosis immunisation experiment at Neuengamme concentration camp was exemplary.^10^ The question arises whether what Schwarberg achieved in microcosm can be achieved for the totality of victims. Our aim is to identify not just clusters of victims but all victims of unethical medical research under National Socialism. The methodology is that of record linkage to reconstruct life histories of the total population of all such research victims. This allows one to place individual survivors and groups of victims within a wider context.

This project on the victims of Nazi medical research represents the fulfilment of Thompson's original scheme of a complete record of all coerced experiments and their victims.^11^ Our project identifies for the first time the victims of Nazi coercive research, and reconstructs their life histories as far as possible. Biographical data found in many different archives and collections is linked to compile a full life history, and subjective narratives and administrative data are compared. Results are aggregated here as cohorts because of undertakings as regards anonymisation, given in order to gain access to key sources. All data is verifiable through the project database.

The criterion for unethical research is whether coercion by researchers was involved, or whether the location was coercive. The project has covered involuntary research in clinical contexts as psychiatric hospitals, incarceration in concentration camps and prisoner of war camps, the ‘euthanasia’ killings of psychiatric patients with subsequent retention of body parts for research, and executions of political victims, when body parts were sent to university anatomical institutes, and persons subjected to anthropological research in coercive and life threatening situations as ghettoes and concentration camps.

**Figure 1:**
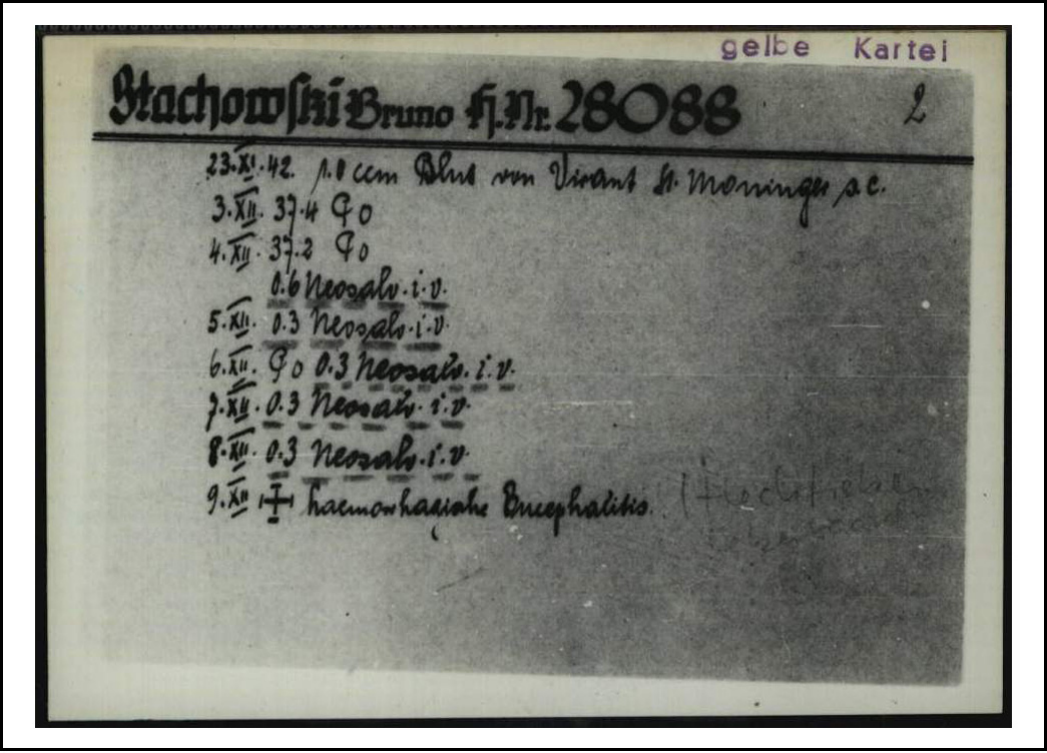
Malaria card of Father Bruno Stachowski from Claus Schilling's research at Dachau. Approximately 1000 cards were kept back from destruction by the prisoner assistant Eugène Ost. International Tracing Service, source number 1079406301.

Without a reliable, evidence-based historical analysis, compensation for surviving victims has involved many problems. Victim numbers have been consistently underestimated from the first compensation scheme in 1951 when the assumption was of only few hundred survivors.^12^ The assumption was that most experiments were fatal. This project's use of several thousand compensation records in countries where victims lived (as Poland) or migrated to (as Israel), or were collected by the United Nations or the German government has corrected this impression. The availability of person-related evidence from the International Tracing Service at Bad Arolsen further helps to determine whether a victim survived. Major repositories of documents like the United States Holocaust Memorial Museum and the Yad Vashem archives, court records in war crimes proceedings, and oral history collections notably the Shoah Foundation have been consulted. Record linkage of named records is essential for the project, and shows how a single person could be the victim of research on multiple occasions. Father Leon Michałowski, born 22 March 1909 in Wąbrzeźno, was subjected to malaria in August 1942 and then to freezing experiments in October 1942 (Figure 1).

A further issue relates to the methods and organisation of the research. From the 1950s the experiments were viewed as ‘pseudo-science’, in effect marginalising them from mainstream science under National Socialism. For the purpose of this study, the experiments have been viewed as part of mainstream German medical research, as this renders rationales and supportive networks historically intelligible. It is clear that prestigious research institutions such as the Kaiser Wilhelm Society and funding agencies such as the German Research Fund were involved.^13^ It has been argued more recently that some experiments were cutting edge science.^14^ Another view is that the approach and methods were scientific albeit of varying quality. For the purpose of this study, the experiments have been viewed as part of mainstream German medical research, as this renders rationales and supportive networks intelligible.

Defining what constitutes research is problematic. For example, a listing of operations in a concentration camp may be nothing more than a clinical record, may have been undertaken by young surgeons seeking to improve their skills, or may indeed have involved research. As stated above, only confirmed data of research has been utilised in the project's category of a verified instance of unethical research. The only exception is the corpses sent to anatomical institutes for research purposes.^15^ Separating these out often does not appear possible, but the project includes anatomical research on body parts and brains as separate categories.

The project has graded victim evidence into two categories, so that there should be a set of verifiable and proven victims established as incontestable evidence of having been a victim. The unexpectedly high numbers of identified experiment victims makes this necessary. The two categories are:

1. those who were identified as confirmed victims through a reliable source such as experimental records kept at the time.
2. those who have claimed to have been experimented on, but confirmation could not so far be obtained.

The project did not set out to adjudicate on the authenticity of victims’ claims. In Warsaw ca. 3600 compensation files of victims of human experiments were viewed, while there are a further 10,000 files representing claims deemed unsuccessful. It is sometimes unclear whether extensive injuries were retrospectively defined to have resulted from an experiment to meet the criteria of the compensation scheme offered by the Federal Republic of Germany in various forms since 1951, or whether experimentation had taken place in a hitherto unknown location. The project discounted claims of abuse when no experiment or research was involved, or when victims having misunderstood compensation schemes for experiments being about ‘experiences’. It is hoped that further research will provide confirmation of experiments in disputed locations like the concentration camps of Stutthof and Theresienstadt.^16^ While Yugoslav victims were abused for experiments in German concentration camps, claims for experiments in the former Yugoslavia and Northern Norway have not so far been confirmed. The grading of victims’ claims into the verified and as yet unverified enable the project to establish verifiable minimum numbers, while indicating the possibility of higher numbers being confirmed by further research.

## Project findings

**Figure 2:**
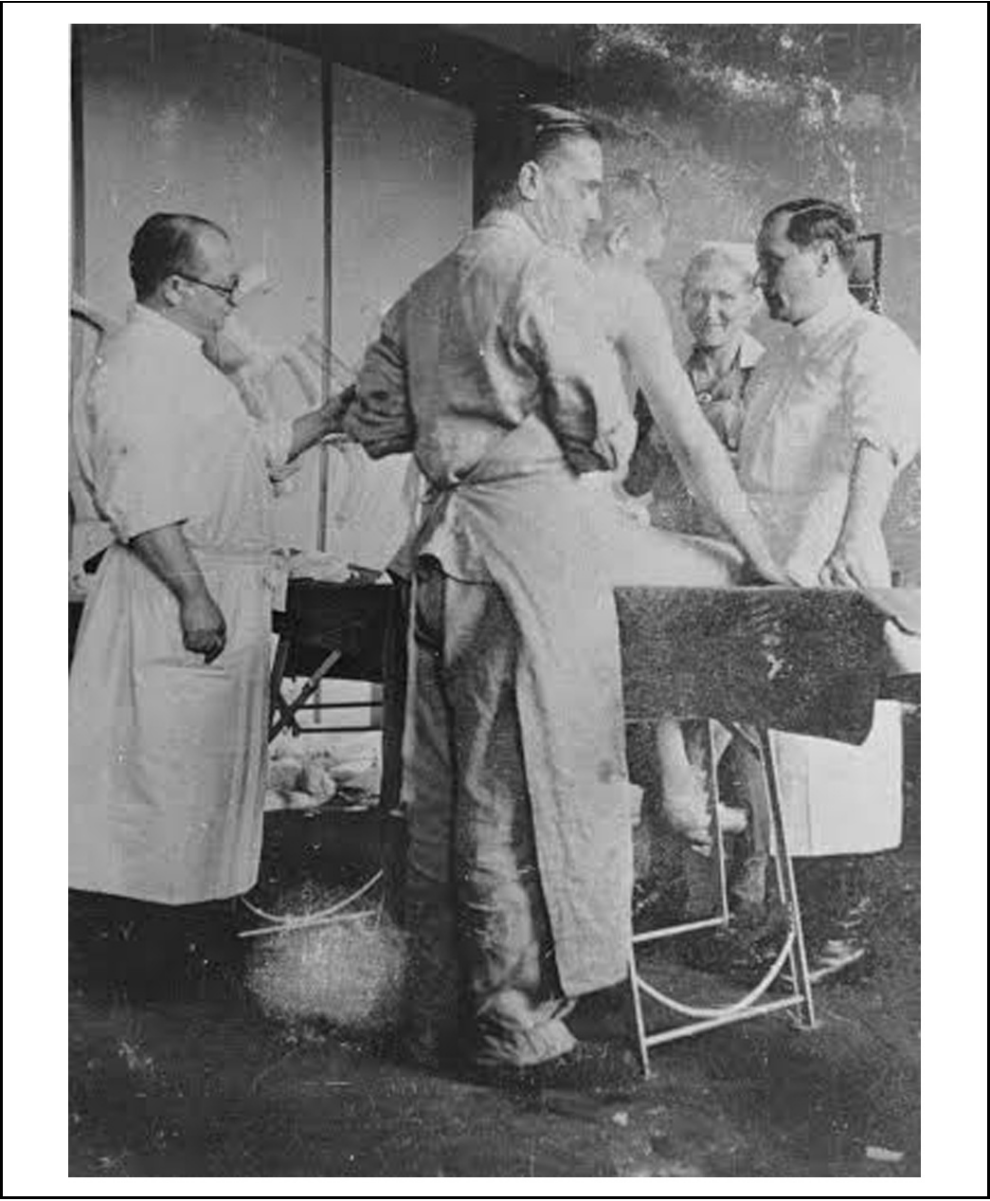
Carl Clauberg and Horst Schumann at Block 10 in Auschwitz. United States Holocaust Memorial Museum W/S #67417.

The project is able to present results on: how many victims were killed in the course of the experiment, how many died from the consequences of the experiment or were killed as potential evidence of Nazi criminality, and how many survived? The project has covered experiments, as the most notorious experiments taken to the point of death and supported by the SS in concentration camps, as well as dispersed experiments in a variety of clinical contexts, particularly on psychiatric patients. Some sets of experiments and locations, not least those sponsored by German pharmaceutical companies remain shadowy, and require more detailed research possibly on the basis of further disclosure of documents held in company archives. The extent of involvement of German pharmaceutical companies like that of IG-Farben (using the branded product names of ‘Bayer’, ‘Hoechst’ and ‘Behringwerke’) remains contentious. The company supplied Helmuth Vetter with samples for experiments at Auschwitz and Mauthausen. Similarly problematic is the extent that Schering-Kahlbaum supported Clauberg's uses of X-ray contrast fluids and a substance to seal the fallopian tubes at Auschwitz. Initially, Clauberg asked for deliveries to his clinic at Königshütte (so making the experiments appear as taking place in a consensual clinical context), but later on to Auschwitz. The extent that the company's senior staff knew that their employee and Clauberg's pharmaceutical assistant Johannes Goebel worked at Auschwitz is contentious (Figure 2).^17^

The occurrence of unethical research provides insight into the structure of Nazi medical research. The project traced how Nazi coercive research began in the context of eugenic research in the mid-1930s. After numbers of experiments dipped in 1940 due to military call-up of medical researchers, the research rapidly intensified both in terms of numbers of experiments and victims, and in terms of severity for victims. This can be seen from 1942 with the notorious and often fatal experiments on low pressure, exposure to freezing temperatures, and infectious diseases when research could be taken to the point of death. Pharmacological experiments on therapies for tetanus, typhus and typhoid were spurred by the realisation that Allied military medical research on infectious diseases was outstripping German military medical expertise. From November 1942 racial priorities came increasingly to the fore, as exemplified by Schumann's X-ray sterilisation experiments on Jews in Auschwitz.

Victims were a highly international group. The above table (Table 1) shows numbers of nationalities, using nationality as in 1938. The table indicates the distribution of nationalities. The largest national group, that of Polish victims, includes both Roman Catholics and Jews. There were high numbers of German and Austrian victims, in part as a result of the experiments and dissections that accompanied the killing of psychiatric patients. While there were other large groups, there are also smaller national groups, as Swiss, British and Irish, all highly remarkable in how their citizens became caught up in the experimentation. We find victims include a Swiss conscientious objector used for malaria experiments at Dachau, and British commandos captured in Norway used for amphetamine and high performance experiments on the shoe track at Sachsenhausen, and subsequently executed.

**Figure 3:**
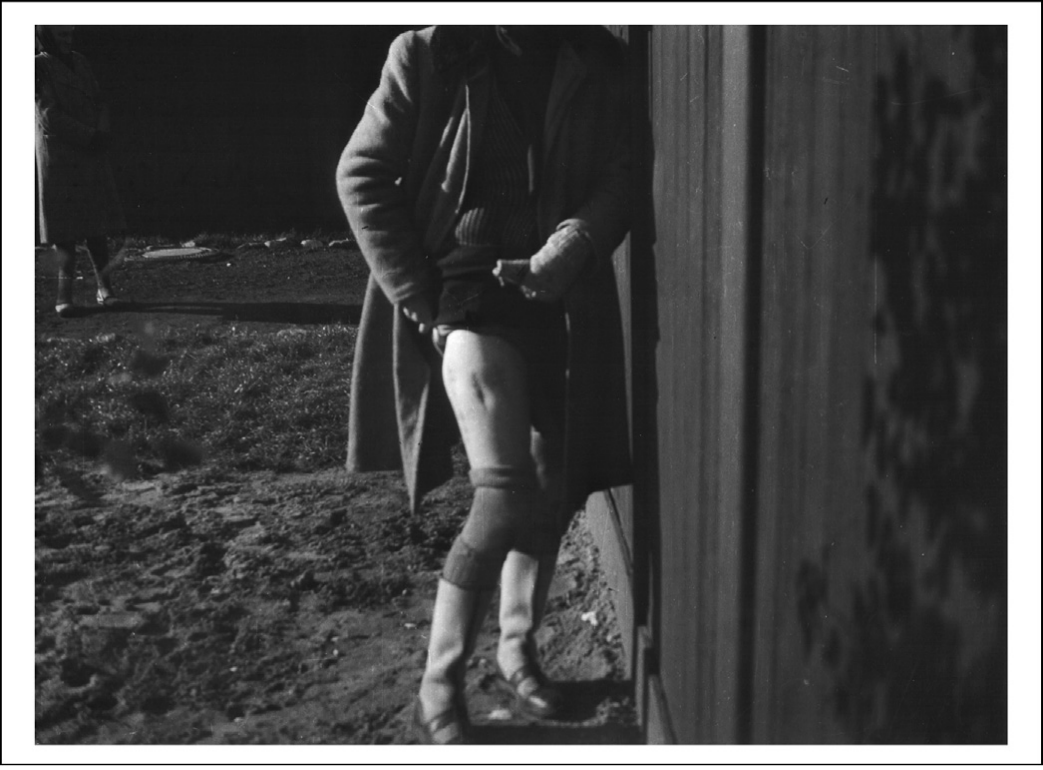
Clandestine photograph of a mutilated leg of the Polish political prisoner Bogumiła Babińska-Dobrowska at Ravensbrück concentration camp. United States Holocaust Memorial Museum, courtesy of Anna Hassa Jarosky and Peter Hassa W/S #69340.

Statistics on gender indicate a proportion of male to female of approximately 2:1 (Table 2). One possible reason is the high number of military experiments as related to infectious diseases. Another is that more men than women were held in concentration camps, so that there was a higher male availability in the predominately male camps. In Ravensbrück the situation was reversed with the large female camp and a small male compound (Figure 3).

While for most nationalities male victims were the majority, in the case of certain national groups, female victims were in the majority. This is the case for victim groups from the Netherlands (in the case of sterilisation at Auschwitz), and Greece (for the Jewish skeleton collection). Children were often victims of experiments in psychiatric clinics. Later in the war, Roma and Jewish children were targeted for research by Mengele in Auschwitz.

**Figure 4:**
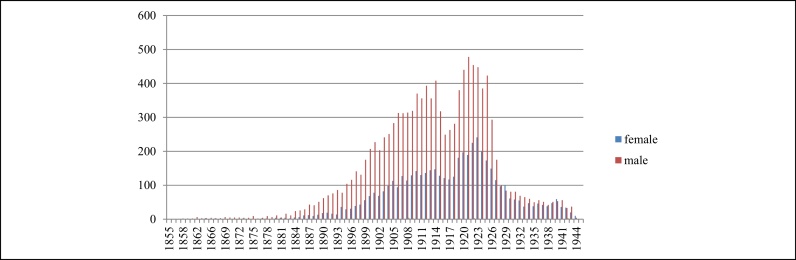
Age of victims at the start of experiment.

The statistics show the age distribution was the same for men and women. While there was a very wide age spectrum, the peak is of victims born in 1921, so in their early twenties at the time of the experiment (Figure 4). Several hundred Jewish children were held by Mengele for twin research, and batches of Jewish children were dispatched for hepatitis and tuberculosis research, and body parts of small children were retained by psychiatric researchers.

Ethnicity and religion have been recorded, as for the definitively confirmed experiment victims (Table 3). Here, one is thrown back on the categories imposed by the Nazis. Thus a victim of the Jewish skeleton collection for the anatomy department at Strasbourg was baptised Protestant.^18^ Generally, the Nazis used the generic and stigmatising term of ‘Zigeuner’ or gypsy rather than the self-identifying terms of ‘Sinti’ and ‘Roma’.

**Figure 5:**
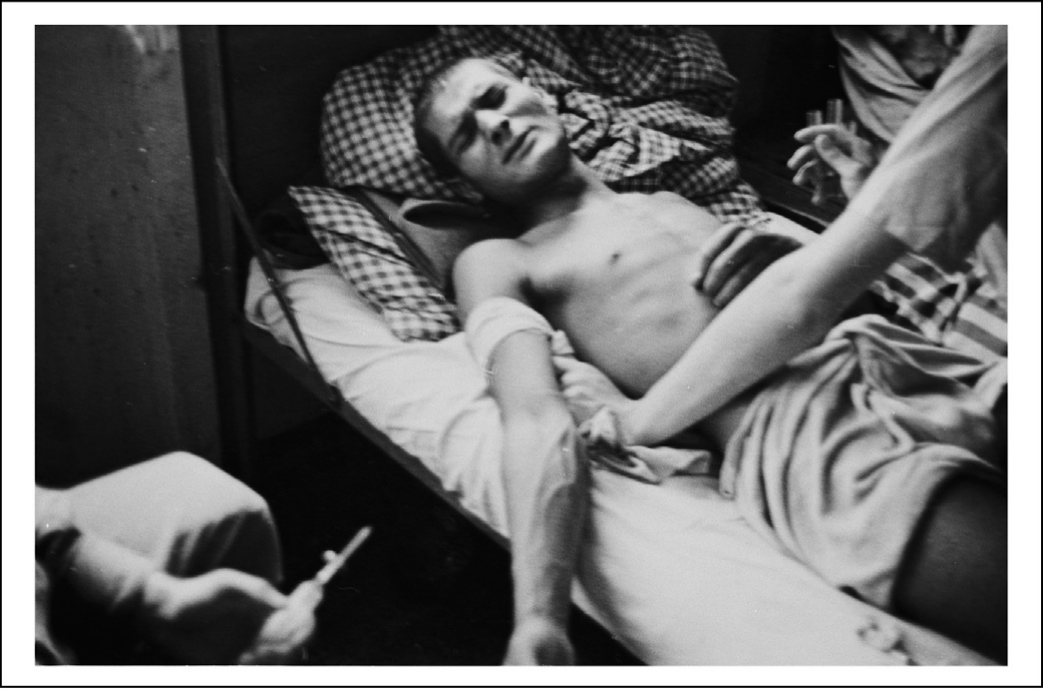
A gypsy used for seawater experiments in Dachau to test methods of making seawater drinkable, ca. July–September 1944. United States Holocaust Memorial Museum, courtesy of National Archives and Records Administration, College Park W/S #78688.

In addition to the experiment victims are Roma and Sinti victims of large scale anthropological investigations of Ritter, Justin, and Ehrhardt, amounting to at least a further 21,498 persons (Figure 5).

If however one takes the year 1943 we find a higher proportion of Jewish victims, in part because of the intensification of experiments on Jews (particularly on women and children) at Auschwitz and Auschwitz-Birkenau. This would again indicate that there was an intensification of racial research (Table 4).

**Figure 6:**
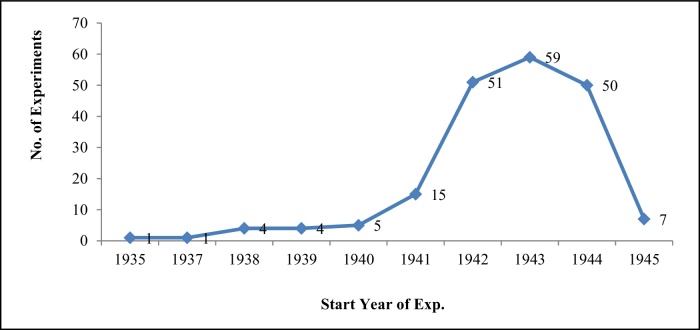
Start year of experiments.

Victim number indicates how from 1942 onwards there was an overall intensification of research (Figure 6).

The life history approach allows appraisal of both experiments and victim numbers over time. The period 1933–39 shows sporadic experimentation in the context of racial hygiene. Mixed race adolescents were sterilised and evaluated by anthropologists. The concerns of racial hygiene with mental illness explain why psychiatrists and neurologists conducted experiments in psychiatric institutions. The psychiatrist Georg Schaltenbrand pointed out that his neurological research subjects were transferred to other institutions, many as we now know to be killed. This interrupted his research on the transmissibility of multiple sclerosis. The numbers of brains and body parts increased. From 1942 onwards there was an overall intensification of research.

**Figure 7:**
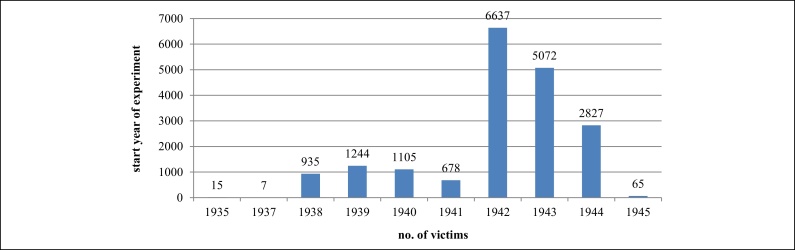
Victims by start year of each experiment.

The chart (Figure 7) shows when experiments started, but not the distribution of victims over time.

The largest series of experiments were for infectious diseases. Malaria research at Dachau between 1942 and 1945 had 1091 confirmed victims, and after infection different combinations of drugs were tested. These experiments by Schilling began in 1942 and remarkably Schilling tried to continue the research after the liberation of the camp.^19^

**Figure 8:**
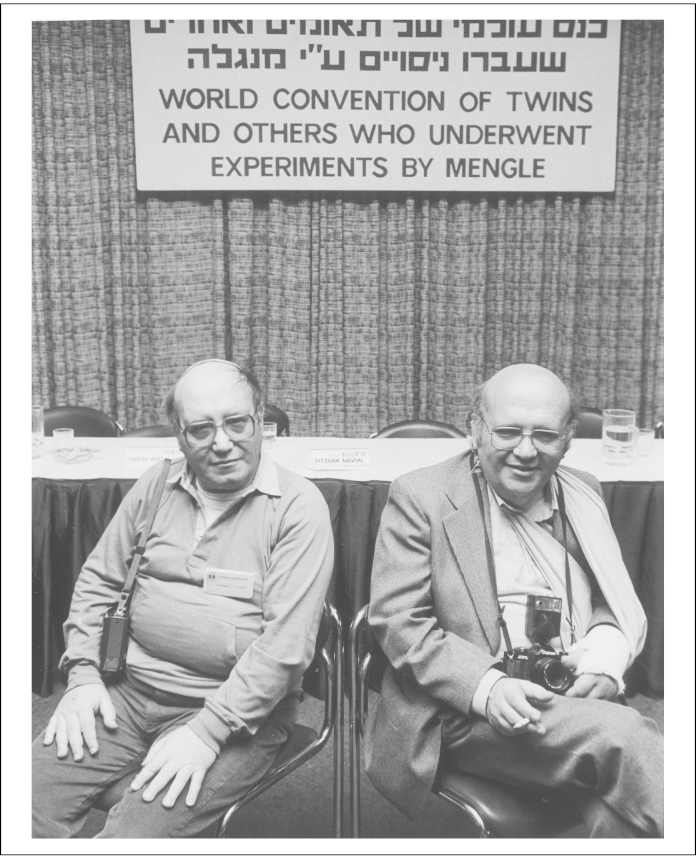
Twins Frank (lt) and Otto (rt) Klein attend a world gathering of survivors of Dr. Joseph Mengele's medical experiments at Auschwitz. United States Holocaust Memorial Museum W/S #05586.

He pleaded at his trial to be allowed to continue the research, albeit on volunteers. The highest numbers were in 1943. The momentum continued even though the war was clearly lost. Other large groups included the twins researched on by Mengele, and to date 618 individuals are known (Figure 8).

The overall findings provide an accurate basis for analysis of experiments to date. First, nearly a quarter of confirmed victims were either killed to obtain their organs for research, or died as a result of experiments taking the research subject to the point of death (notoriously, the experiments on freezing and low pressure at Dachau). The euthanasia killings and executions were sources of bodies for research, and the extent that this happened and research conducted before and after the end of the war is still being documented. Of the fully documented victims died 781 died before the end of the war as a result of the experiments: research subjects were weakened by the strain of the experiment such as a deliberate infection or severe cold, or they were deliberately killed because it was feared that they would testify against the perpetrators (Table 5).

While, most subjects survived, amounting to 24,010 persons, many had severe physical disabilities with life-long consequences.^20^

## Conclusion

The analysis presented here shows that several types of unethical medical research occurred under National Socialism. Not only were large numbers of victims affected, but also overall, numbers of surviving victims were far higher than anticipated. The survivors were often seriously disabled and handicapped for the remainder of their lives. The experiments gained in numbers with the war and the implementation of the Holocaust, and were sustained at a high level of intensity despite imminent defeat.

One issue arising is that body parts of deceased victims were retained by medical research and teaching institutes, notably for anatomy and brain research. While there was meant to be full disclosure of specimens deriving from euthanasia victims and executed persons by 1990, specimens continue to be identified.^21^ The complex data is to be further augmented and refined, the history of specimens retained for research during and after WW2 is being documented, and the narratives of survivors analysed in order to understand more fully the consequences of coerced research. This research provides a basis in historical evidence for discussions of the ethics of coerced medical research.

## Figures and Tables

**Table 1:** Nationality (as at March 1938).

Nationality	Confirmed victim	Pending	Total
Austrian	782	17	799
Belgian	16	32	48
British	16	2	18
Czechoslovakian	264	1020	1284
Danish	2	1	3
Dutch	265	26	291
French	156	57	213
German	2254	123	2377
Greek	426	18	444
Hungarian	609	1393	2002
Irish (Republic)	1		1
Italian	71	6	77
Latvian	1	1	2
Lithuanian	4	2	6
Luxembourgian	1		1
Norwegian	11	1	12
Polish	2737	4168	6905
Romanian	51	39	90
Soviet	1022	26	1048
Spanish	22	4	26
Stateless	449	4	453
Swedish	1		1
Swiss	3		3
Yugoslav	536	3421	3957
Unknown	6054	1644	7698

Grand total	15,754	12,005	27,759

**Table 2:** Gender.

Gender	Confirmed victim	Pending	Total
Female	3960	4381	8341
Male	9700	7188	16,888
Unknown	2094	436	2530

Total	15,754	12,005	27,759

**Table 3:** Ethnicity.

Ethnicity	Confirmed victims (15,754)
Jewish	20% (3098)
Roma and Sinti	2% (335)
Unknown or other	78% (12,321)

**Table 4:** Religion.

Religion	Confirmed victims	Pending	Total
Jewish	3076	792	3868
Other or unknown^a^	12,678	11,217	23,891

Grand total	15,754	12,008	27,759

a This category includes: Christians (Catholics, Protestants, and Orthodox), Muslims, Jehovah's Witnesses, Seventh-day Adventists and atheists.

**Table 5:** Fatalities.

Circumstances of death	Confirmed victim	Pending	Total
Body used for research (e.g. euthanasia and executed victims)	2956	50	3006
Died (e.g. from injuries) or killed after the experiment	781	23	804
Died from experimental procedures (e.g. when onset of death studied from freezing)	383	171	554

Grand total	4120	244	4364

## Footnotes

7 Annas G, Grodin M. *The Nazi Doctors and the Nuremberg Code*. New York: Oxford University Press; 1992; Weindling P. *Nazi Medicine and the Nuremberg Trials: From Medical War Crimes to Informed Consent*. Basingstoke: Palgrave Macmillan; 2000.

8 Weindling P. *John Thompson (1906–1965): Psychiatrist in the Shadow of the Holocaust*. Rochester: Rochester University Press; 2010.

9 Klee E. *Auschwitz, die NS-Medizin und ihre Opfer*. Frankfurt am Main: S. Fischer Verlag; 1997; Mitscherlich A, Mielke F. *Wissenschaft ohne Menschlichkeit*. Heidelberg: Lambert Schneider; 1949. See also Schwarberg G. *The Murders at Bullenhuser Damm: The SS Doctor and the Children*. Bloomington: Indiana University Press; 1984.

10 Lang H-J. *Die Namen der Nummern: Wie es gelang, die 86 Opfer eines NS-Verbrechens zu identifizieren.* Hamburg: Hoffmann und Campe; 2004.

11 Brody H, Leonard S, Nie J-B, Weindling P. United States Responses to Japanese Wartime Inhuman Experimentation after World War II: National Security and Wartime Exigency. *Cambridge Quarterly of Healthcare Ethics* 2014; 23: 220–230.

12 Baumann S. *Menschenversuche und Wiedergutmachung*. München: Oldenbourg; 2009.

13 Schwerin A von. *Experimentalisierung des Menschen: Der Genetiker Hans Nachtsheim und die vergleichende Erbpathologie 1920–1945*. Göttingen: Wallstein Verlag; 2004; Schmuhl H-W. *The Kaiser Wilhelm Institute for Anthropology, Human Heredity and Eugenics, 1927–1945: Crossing Boundaries*. Dordrecht: Springer; 2008.

14 Roelcke V. Fortschritt ohne Rücksicht, In: Eschebach I, Ley A. eds*. Geschlecht und “Rasse” in der NS-Medizin*. Berlin: Metropol Verlag; 2012: 101–114.

15 Hildebrandt S. Anatomy in the Third Reich: An outline, Part 1. National Socialist politics, anatomical institutions, and anatomists. *Clinical Anatomy* 2009; 22: 883–893.

16 Hunt N. *The Soviet Experience of Nazi Medicine: Statistics, Stories and Stereotypes*. MPhil thesis, Oxfrod Brookes University; 2011; and Loewenau A. *The impact of Nazi medical experiments on Polish inmates at Dachau, Auschwitz and Ravensbück*. PhD thesis, Oxford Brookes University; 2012.

17 Weindling, P. *Victims and Survivors of Nazi Human Experiments: Science and suffering in the Holocaust.* London: Bloomsbury; 2014.

18 www.die-namen-der-nummern.de/html/the_names.html (accessed 8 October 2014).

19 Eckart WU, Vondra H. Malaria and World War II: German malaria experiments 1939–45*. Parassitologia* 2000; 42:53–58.

20 Loewenau A. Die “Kaninchen” von Ravensbrück: Eine Fotogeschichte. In: Eschebach I, Ley A eds. *Geschlecht und “Rasse” in der NS-Medizin*. Berlin: Metropol Verlag; 2012:115–140.

21 Weindling P. “Cleansing” Anatomical Collections: The Politics of Removing Specimens from German Anatomical and Medical Collections 1988–92. *Annals of Anatomy* 2012; 194:237–242.

